# Is Endemicity a Solution for the COVID-19 Pandemic? The Four E's Strategy for the Public Health Leadership

**DOI:** 10.3389/fpubh.2022.911029

**Published:** 2022-06-28

**Authors:** Leonardo Villani, Maria Rosaria Gualano, Walter Ricciardi

**Affiliations:** ^1^Section of Hygiene, University Department of Life Sciences and Public Health – Università Cattolica del Sacro Cuore, Rome, Italy; ^2^Department of Public Health Sciences, University of Turin, Turin, Italy

**Keywords:** COVID-19, endemicity, leadership, health inequalities, infectious diseases

## Introduction

In recent times the debate about the possibility, and in some cases the hope, that the COVID-19 disease will become endemic has gained momentum. Indeed, in some cases, public opinion and scientists have proposed and enthusiastically welcomed this evolution, indicating a change of phase of the pandemic and a possible resolution of it.

Infectious diseases can evolve in four scenarios, in relation to their biological characteristics (mutations that arise as a result of specific selective pressures might determine the occurrence of variants) and the Public Health measures implemented to contain the spread of the pathogen. Incidence, prevalence and geographical distribution of the disease, in fact, identify the conditions of extinction (the pathogen no longer exits, both in nature and in the laboratory, on a global scale), eradication (permanent zero incidence globally, so as not to require further interventions of Public Health: there is no risk of reappearance of the disease), elimination of the pathogen and the disease (zero incidence in specific geographic areas, following continuous Public Health interventions) and endemicity (constant presence and/or habitual prevalence of an infectious agent in a population within a geographic area with coexistence between human and the pathogen) (1, 2). The latter, therefore, requires a constant control of the epidemiological trend of the disease, in order to maintain acceptable levels of incidence, prevalence and mortality. While there are no examples of extinction, smallpox, and rinderpest represent examples of eradication, while polio and measles of elimination.

That stated, is the evolution toward endemicity really desirable with a disease with high transmission and mortality rates such as the COVID-19? is the evolution toward endemicity really desirable? In this context, it is important to point out a few considerations about the health, social and economic burden of endemicity and the possible impacts of COVID-19 endemicity, summed up in the 4 E's strategy, as follows.

## The 4 E's Strategy

### Estimating the Health and Healthcare Services Impact of Endemicity

Endemic infectious diseases have a huge health impact, as they are responsible for the deaths of millions of people each year. Tuberculosis and malaria, for example, as well as HIV/AIDS, are responsible of about 1.5 million, 600,000, and 700,000 deaths each year, respectively, with high mortality rates (about 20/100,000, 15.3/100,000, and 10/100,000, respectively) (3–5). By comparison, in 2021, there were about 3.5 million deaths due to COVID-19 worldwide with a mortality rate of about 50/100,000, despite the spread of vaccines and the availability of new diagnostic and therapeutic tools.

Moreover, endemic diseases (such as malaria, tuberculosis, and AIDS) have an important impact on the quality of life of people, and can lead to the development of chronic conditions, reducing life expectancy and increasing the years lived with disability, so as to be among the top leading causes of Disability-adjusted life year (DALYs) (6). Increasing literature is showing the impact of COVID-19 in terms of DALYs (7, 8) and reduction of life expectancy (9, 10), with a burden that might persist and worsen in the coming years. Likewise, the long-term effects of the COVID-19 are partially known, although early evidence from long-COVID are associated with the persistence of more than 50 clinical conditions in patients (11). This may have a huge impact on population health in long-term period, with an important health, social and economic burden on health systems. These considerations are even more true if we consider the pediatric age: it is well-established that infectious diseases (i.e., pneumonia) contracted in childhood or adolescence might have important sequelae on organ function in adult life (12) and the same pattern could be observed in COVID-19 pediatric patients (13). In addition, the healthcare services will continue to have a large number of patients to assist in the next years, since the burden of the disease continue to exist. In this context, many countries made a great effort by increasing healthcare spending in order to provide more beds, medical personnel, drugs and technologies needed to counter the pandemic. However, this effort may not be sustainable in the future, and in any case, it may decrease attention toward the management of other diseases (14). Indeed, the pandemic caused and is still causing a disruption in all healthcare settings in both low- and high-income countries, increasing the burden of other diseases, especially chronic degenerative and oncological, with a delay in diagnosis and treatment, making people unable to access care at the primary care and community care levels (15).

### Encountering the Social Impact of Endemicity

Endemic infectious diseases have a devastating impact in social terms, causing negative consequences both at individual (divorces, low household income and poverty, stigmatization, social exclusion) and country level (permanent condition of poverty, reduced economic growth, and discouraging investments and tourism) (16–18).

The COVID-19 pandemic has resulted in school closures, disruption of education, and interruption of social and recreational activities (19). These closures, necessary to contain the spread of the virus, have and will have a devastating impact on people's mental health, especially children, and adolescents (20, 21). The trend toward an endemic condition, which does not exclude the appearance of new epidemic waves, as happened with influenza viruses (22), could lead to new closures, with unacceptable damage to the population. In this context, the persistence of the virus in the population guarantees the spread of variants, whose evolution in terms of lethality and transmission capacity cannot be predicted (23). Thus, it is important to take caution and remember that the pandemic is still ongoing. Maintaining public health containment measures (hand hygiene, proper ventilation of rooms, mask use, and physical distancing) are important conditions that should not be avoided. At the same time, it will be necessary to ensure social recovery mechanisms and investment in order to prevent the immediate and long-term impact of the pandemic on wellbeing, poverty, and the onset of inequality (24).

### Evaluating the Economic Impact of Endemicity

Endemic infectious disease has a significant economic impact, both in terms of direct (personal and public expenditures on both prevention and treatment of the disease) and indirect costs (lost productivity associated with illness or death). For example, it is estimated that tuberculosis will have a cost of about 1 trillion USD in the period 2015–2030 (25), while the global cost of malaria is estimated of about 12 billion USD per year (26). Moreover, the economic impact can be observed in countries with endemic diseases that remain in a condition of poverty and reduced economic growth, contributing to lifelong disadvantage in an already disadvantaged group and establishing a vicious circle from which it is difficult to find a way out (27).

Considering the COVID-19, in 2020 the pandemic resulted in a contraction of global GDP of 3.2%, with a projected cumulative output loss during 2020 and 2021 of about USD 8.5 trillion (28) with a slow economic recovery for the next years (29). Moreover, COVID-19 has an important impact in terms of costs related to healthcare assistance: indeed, in the US it was estimated a total cost that range between about USD 11,000 and 47,000 per hospitalization (30), and same results are reported in Europe (31, 32), highlighting that it can be particularly difficult to address these costs in all health services, especially universal health services.

### Enhancing the Attention of Endemicity Impacts in Low-Income Countries

Endemic diseases, such as tuberculosis and malaria, often remain in the most disadvantaged areas of the world (such as African countries), which have reduced access to care and treatment and vaccination. Thus, there is a real risk that the evolution toward endemicity will be borne more by low-income countries, where an additional serious disease would be added. The most disadvantaged areas of the world are currently unable to cope with diseases that are already present. It is therefore essential to prevent a new disease from becoming endemic in these territories. This condition would further increase the economic, social, and health burden on countries already severely damaged by these diseases. In particular, although in Africa the reported prevalence of the SARS-CoV-2 is lower than expected, it is necessary to carefully consider possible explanations for this evidence: while it could be due to the presence of other diseases and related therapies in use in these countries (33), it should be noted that the epidemiologic surveillance systems in these countries are weak, and therefore a strong underestimation of the number of cases and deaths is possible (34–37), with an estimated 97 times as many confirmed cases as reported (38). Moreover, drugs used in malaria prophylaxis, such as hydroxychloroquine, have been shown to be ineffective and even harmful in the treatment of COVID-19, thus ruling out a possible protective action of such drugs (39, 40). In addition, the absence of structured epidemiological surveillance systems is associated with lack of professional skills and capacities, absence of facilities, diagnostic tools, and the presence of other epidemics to be monitored, which make it difficult to manage the pandemic, in addition to the difficulties in reaching patients living in rural areas (41).

## Discussion

Are we really willing to accept the evolution of the disease toward endemicity with optimism? And above all, is it really right, in terms of Public Health, to favor this evolution? The tools to contain the infection, such as diagnostic tools, vaccines and specific drugs, are available: it is therefore necessary a strong international leadership that can really lead the fight against the virus, through three key actions.

First, it is necessary making treatments and vaccines available to all. Equity in access to treatment and vaccines is and must be a priority for all in order to counteract the trend toward endemicity and facilitate the conclusion of the pandemic. Currently, only 15% of the population living in low-income countries is vaccinated, against an average of 70/80% in high-income countries (42). These data reflect the accessibility to vaccination: in high-income countries, in fact, although the availability of COVID-19 vaccines, ignorance, miscommunication, and in some cases the absence of a strong central leadership that follow the scientific evidence, have caused vaccine hesitancy (43, 44). On the contrary, in low-income countries there is a lack of vaccines due to several aspects such as the absence of infrastructure and technology for vaccines production and maintenance, and the problem of the suspension of patent protections (45). In this context, despite the commitment made by the high-income countries and the World Health Organization (46), it could be difficult to ensure a total and above all continuous vaccination campaign (i.e., with booster doses) (47).

Moreover, in addition to vaccine availability worldwide, it is important to provide vaccine updates as frequent SARS-CoV-2 mutations are decreasing vaccine efficacy (48), always considering that vaccination, for both COVID-19 and other types of diseases, is one of the most cost-effectiveness intervention in healthcare, even in relation to high need for doses (49–51).

Second, create strong international scientific leadership that can guide, and direct government choices based on scientific evidence. In particular, multidisciplinary scientific research contributed enormously to the rapid identification of drugs and vaccines, as well as providing evidence about the mechanisms of action of the virus and consequently also of the effectiveness of containment measures. Basic research, clinical trials and epidemiological studies have helped to expand knowledge about COVID-19 with unprecedented rapidity (52–54). However, in some cases policymakers, discouraged from making unpopular decisions, have ignored scientific evidence, with devastating effects in their countries (55–57). Thus, albeit science cannot replace the integrity of public leadership, it is necessary to build bridges between research and politics in order to cooperate and support policy decisions and help policymakers make unpopular decisions, increasing people's confidence in science and politics (58, 59). In this context, it is worth noting the apparent incongruence between science, based on evidence, and politics, which is required to take swift action during emergency situations. Evidence-based medicine is a lengthy process derived from the sum of the knowledge's, so it may be difficult to apply to new emergency situations. However, it is possible to rethink it in these kinds of settings, recognizing, for example, the role of experts and fostering their involvement and cooperation in policy decision making (58, 60), including through the creation of national and international agencies, such as the new European Health Emergency Preparedness and Response Authority (HERA) (61). In this way, scientists can provide important policy decision support, based, in the absence of solid evidence, on appropriateness, reasonableness, and evidence from similar contexts.

Third, there is a need to work on the education and cultural aspects of the population, and, often, of policymakers. Mistrust in science, which has become more acute in some segments of the population in some countries, represents a major issue in the management of both the current and future emergencies. This condition might be fixed by a univocal communication adherent to scientific evidence at international and national institutional level, which has often been lacking during the pandemic (the management of communication about Vaxzevria adverse reactions is a cogent example).

In conclusion, although the epidemiological evolution shows a trend toward endemicity, it is necessary to make public opinion and policymakers understand that this may have significant long-term effects in health, social, and economic terms. It is therefore necessary to increase the commitment to ensure vaccination at the global level (with the production of increasingly specific, updated, and effective vaccines), which currently represents the strongest tool to contain the spread and severe symptoms of the disease. On one hand the example of diseases of the past (smallpox and rinderpest) show how the eradication of the virus is possible, on the other endemic diseases show the huge burden at global level and especially in low-income countries. Public health has the opportunity and the capacities to support governments in their policy activities and to advocate for evidence-based strategies at national and international levels to build a common front in response to the pandemic: let's use them, not give in to endemicity.

## Author Contributions

All authors listed have made a substantial, direct, and intellectual contribution to the work and approved it for publication.

## Conflict of Interest

The authors declare that the research was conducted in the absence of any commercial or financial relationships that could be construed as a potential conflict of interest.

## Publisher's Note

All claims expressed in this article are solely those of the authors and do not necessarily represent those of their affiliated organizations, or those of the publisher, the editors and the reviewers. Any product that may be evaluated in this article, or claim that may be made by its manufacturer, is not guaranteed or endorsed by the publisher.
